# Multivariate normative comparisons using an aggregated database

**DOI:** 10.1371/journal.pone.0173218

**Published:** 2017-03-07

**Authors:** Joost A. Agelink van Rentergem, Jaap M. J. Murre, Hilde M. Huizenga

**Affiliations:** 1 Department of Psychology, University of Amsterdam, Amsterdam, The Netherlands; 2 Amsterdam Brain and Cognition Center Amsterdam, University of Amsterdam, Amsterdam, The Netherlands; 3 Research Priority Area Yield, University of Amsterdam, Amsterdam, The Netherlands; University of Nottingham, UNITED KINGDOM

## Abstract

In multivariate normative comparisons, a patient’s profile of test scores is compared to those in a normative sample. Recently, it has been shown that these multivariate normative comparisons enhance the sensitivity of neuropsychological assessment. However, multivariate normative comparisons require multivariate normative data, which are often unavailable. In this paper, we show how a multivariate normative database can be constructed by combining healthy control group data from published neuropsychological studies. We show that three issues should be addressed to construct a multivariate normative database. First, the database may have a multilevel structure, with participants nested within studies. Second, not all tests are administered in every study, so many data may be missing. Third, a patient should be compared to controls of similar age, gender and educational background rather than to the entire normative sample. To address these issues, we propose a multilevel approach for multivariate normative comparisons that accounts for missing data and includes covariates for age, gender and educational background. Simulations show that this approach controls the number of false positives and has high sensitivity to detect genuine deviations from the norm. An empirical example is provided. Implications for other domains than neuropsychology are also discussed. To facilitate broader adoption of these methods, we provide code implementing the entire analysis in the open source software package *R*.

## Introduction

In neuropsychological assessments, a battery of tests is administered to a patient to determine whether his or her cognitive functions are impaired [1, 2]. Tests within these batteries are designed to assess the patient’s memory, attention, language capacities or other functions. To interpret the patient’s scores, these scores have to be compared to the distribution of test scores in healthy controls. Such a comparison is called a normative comparison. A clinical neuropsychologist may use one standard deviation below the mean as a criterion for impairment [3]. When a patient’s test scores are found to be below normal, this helps the neuropsychologist characterize the patient’s cognitive deficit, and may guide differential diagnosis and treatment.

In neuropsychological research, normative comparisons can be used in a similar way. For example, if a patient and a control group are studied, normative comparisons can be made for each patient in the patient group, with the distribution of test scores in the control group as the reference. In this manner, new variables can be constructed that index whether patients deviate from the norm or not. Such indices may for example be used to assess whether a new treatment, as compared to a waiting list condition, reduces the number of patients who deviate from the norm [4].

Normative comparisons are generally conducted for each test separately: The patient’s test score is compared to the distribution of test scores for that specific test. This is the univariate approach to normative comparisons. An alternative approach is to compare the patient’s profile of test scores to the multivariate distribution of test scores. This is the multivariate approach to normative comparisons [5–8]. Multivariate comparisons have been shown to be more sensitive than univariate comparisons to detect deviations [9]. For example, profiles of high scores on some tests and low scores on other tests, or profiles with many scores that are only a little below normal, are readily detected [7]. An additional advantage is that no correction for multiple comparisons is required [10], because only a single multivariate comparison is conducted. Multivariate normative comparisons have been applied in the study of disorders as diverse as Parkinson’s disease [11–13], stroke [14], prosopagnosia [15], bacterial meningitis [16] and HIV-associated neurocognitive disorder [9, 17].

**Fig 1:**
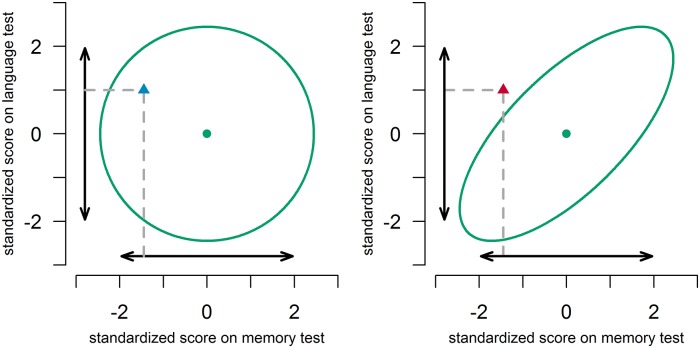
Illustration of multivariate normative comparisons in a situation with scores on two neuropsychological tests. The double-headed arrows denote the 95% univariate intervals. The ellipses denote the 95% multivariate region. The dots denote the mean score in the norm group. The triangles depict a patient’s scores. In the left panel, tests are uncorrelated (r = 0.0). In the right panel, tests are correlated (r = 0.7).

Multivariate normative comparisons for two hypothetical situations are illustrated in Fig 1. In the left panel, the correlation between the memory test score and language test score is 0. In the right panel, the correlation is 0.7. Univariately, the test scores of a hypothetical patient do not deviate, in both panels. Multivariately, the combination of the above average score on the language test, and the below average score on the memory test does not deviate in the left panel, but does deviate in the right panel. In other words, in the right panel, the multivariate comparison shows that the memory score is indeed weak, given the strength of the language score. An experienced clinician may recognize this deviating profile given his/her intuition on the correlation between test scores in the norm group. He/she may be able to decide without using a formal multivariate procedure that the low score on one test together with the high score on the other test is a cause for concern. However, in situations with more than two tests, or situations that are less familiar to the clinician, such decisions will become more difficult. A formal multivariate comparison should then fare better than an informal one, and is likely to promote more accurate diagnostic decisions.

An important drawback of this multivariate method is that multivariate normative data are required, because it is necessary to estimate the covariance of test scores within the norm group [7, 8]. As test developers typically focus on one test, or at most a few tests at a time, these multivariate normative data are not often available. A solution might be to obtain normative data from a neuropsychological study in which a clinical sample has been compared to a healthy control sample on multiple neuropsychological tests. However, a single neuropsychological study will not provide normative data on all neuropsychological tests as, in any single study, only a limited number of tests are administered. Fortunately, by combining data from multiple neuropsychological studies, a dataset can be established that provides all required information. This is the approach that was chosen in a recently started project (www.andi.nl). In this project, a composite normative dataset has been constructed from healthy control data provided by several research institutes. In the following we outline the issues that arise in the construction of such a database.

First, test scores may differ from study to study. Although neuropsychological tests are highly standardized, subtle differences between studies may arise due to the design of the studies. Such differences might for example be caused by differences in incentives that are given to participants, or by differences in the order of test administration. Second, certain tests are administered in one study but not in others (cf. Table 1). That is, for many participants, data will be missing on those tests that were not administered in the study they participated in. The common approach of listwise deletion discards all participants with incomplete data [18], and would result in no participants at all.

**Table 1:** Example of a missing data pattern, where 1 = available, and 0 = missing. For each test, and each study, there are scores missing, although all test co-occur at least once.

	Test 1	Test 2	Test 3
**Study 1**	1	1	0
**Study 2**	1	0	1
**Study 3**	0	1	1

These two issues, missing data and differences between studies, can adequately be handled by multilevel modeling. Multilevel modeling can account for variance between studies [19] and multilevel modeling allows for missing values [20]. Therefore, the present paper provides a multilevel modeling extension of the multivariate approach to normative comparisons.

In making normative comparisons, it is important to correct for background variables that might influence scores. For instance, age may affect reaction times in such a way that a reaction time that implies brain damage in young adults may not be particularly uncommon in a very senior but healthy population. Similarly, a score that implies mild cognitive impairment in highly educated individuals may not be uncommon in healthy individuals with a lower education. Gender usually is less influential, but can make a difference in certain verbal tests, on which women do slightly better than men, and in some visuospatial tests, on which women may do slightly worse [1]. Because of the importance of these background variables, test manuals often contain extensive norm tables to which the score of a patient can be compared. For every background variable that is added as a potential predictor, a new dimension is added to the table.

As an alternative to norm tables that are split for different background variables, regression-based norms are becoming increasingly common [21, 22]. Instead of defining subgroups, participants are compared to the predicted score of a regression equation, in which test scores are regressed on background variables such as age, gender and educational background [23, 24]. In order to correct for these background variables in a regression-based manner, we add the background variables to the multilevel procedure as well.

In this paper, we first describe the multilevel approach to multivariate normative comparisons. We then use Monte Carlo simulations to test the efficacy of this approach in terms of false positives and in terms of sensitivity to genuine deviations from the norm. We demonstrate the application of the method. We conclude by discussing assumptions and by suggesting some future directions.

## Methods

A multilevel analysis requires that the data are structured such that every row of the dataset represents a single test score for one participant. An example with simulated data for three tests is given in Table 2.

**Table 2:** Simulated example of a multilevel dataset with one row per test score. *study* indicates study number; *ID* indicates participant number; *age*, *gender* and *education* are background variables; *z*(1), *z*(2) and *z*(3) are indicator variables; *test* indicates test number and *score* indicates the score on the test with that number.

*study*	*ID*	*age*	*gender*	*education*	*z*(1)	*z*(2)	*z*(3)	*test*	*score*
1	1	−2.21	−1	3.68	1	0	0	1	0.08
1	1	−2.21	−1	3.68	0	1	0	2	1.59
1	2	22.79	1	−0.32	1	0	0	1	0.72
1	2	22.79	1	−0.32	0	1	0	2	2.06
2	1	−25.21	1	0.68	0	1	0	2	0.19
2	1	−25.21	1	0.68	0	0	1	3	1.26
2	2	−11.21	1	1.68	0	1	0	2	0.04
2	2	−11.21	1	1.68	0	0	1	3	−0.29
2	3	3.79	−1	0.68	0	1	0	2	−0.65
2	3	3.79	−1	0.68	0	0	1	3	−0.51

In Table 3, the model specification is given. The model consists of three levels: the level of test scores (abbreviated to tests, although some tests may produce multiple scores), the level of participants and the level of studies.

**Table 3:** Model specification for a multilevel model with three tests, and three background variables (age, gender and level of education), including specification of between and within study covariance structures.

**Level 1 (test: *i*)**							
			*y*_*ijk*_ = *β*_1*jk*_*z*(1)_*ijk*_ + *β*_2*jk*_*z*(2)_*ijk*_ + *β*_3*jk*_*z*(3)_*ijk*_				
**Level 2 (person: *j*)**							
			*β*_1*jk*_ = *ϕ*_10*k*_ + *ϕ*_11*k*_*age*_*jk*_ + *ϕ*_12*k*_*gender*_*jk*_ + *ϕ*_13*k*_*education*_*jk*_ + *ϵ*_1*jk*_ *β*_2*jk*_ = *ϕ*_20*k*_ + *ϕ*_21*k*_*age*_*jk*_ + *ϕ*_22*k*_*gender*_*jk*_ + *ϕ*_23*k*_*education*_*jk*_ + *ϵ*_2*jk*_ *β*_3*jk*_ = *ϕ*_30*k*_ + *ϕ*_31*k*_*age*_*jk*_ + *ϕ*_32*k*_*gender*_*jk*_ + *ϕ*_33*k*_*education*_*jk*_ + *ϵ*_3*jk*_				
**Level 3 (study: *k*)**			**Intercept**		**Age**	**Gender**	**Education**
			*ϕ*_10*k*_ = *γ*_100_ + *ν*_10*k*_		*ϕ*_11*k*_ = *γ*_110_	*ϕ*_12*k*_ = *γ*_120_	*ϕ*_13*k*_ = *γ*_130_
			*ϕ*_20*k*_ = *γ*_200_ + *ν*_20*k*_		*ϕ*_21*k*_ = *γ*_210_	*ϕ*_22*k*_ = *γ*_220_	*ϕ*_23*k*_ = *γ*_230_
			*ϕ*_30*k*_ = *γ*_300_ + *ν*_30*k*_		*ϕ*_31*k*_ = *γ*_310_	*ϕ*_32*k*_ = *γ*_320_	*ϕ*_33*k*_ = *γ*_330_
**Combined (substitution of level 3 into 2, and level 2 into 1)**							
*y*_*ijk*_ =	(*γ*_100_ + *γ*_110_*age*_*jk*_ + *γ*_120_*gender*_*jk*_ + *γ*_130_*education*_*jk*_ + *ν*_10*k*_ + *ϵ*_1*jk*_)*z*(1)_*ijk*_ + (*γ*_200_ + *γ*_210_*age*_*jk*_ + *γ*_220_*gender*_*jk*_ + *γ*_230_*education*_*jk*_ + *ν*_20*k*_ + *ϵ*_2*jk*_)*z*(2)_*ijk*_ + (*γ*_300_ + *γ*_310_*age*_*jk*_ + *γ*_320_*gender*_*jk*_ + *γ*_330_*education*_*jk*_ + *ν*_30*k*_ + *ϵ*_3*jk*_)*z*(3)_*ijk*_						
**Covariance Matrix Within**				**Covariance Matrix Between**			
	Test A	Test B	Test C		Test A	Test B	Test C
Test A	*var*_*ϵ*_1*jk*__			Test A	*var*_*ν*_10*k*__		
Test B	*cov*_*ϵ*_2*jk*_, *ϵ*_1*jk*__	*var*_*e*_2*jk*__		Test B	0	*var*_*ν*_20*k*__	
Test C	*cov*_*ϵ*_3*jk*_, *ϵ*_1*jk*__	*cov*_*ϵ*_3*jk*_, *ϵ*_2*jk*__	*var*_*ϵ*_3*jk*__	Test C	0	0	*var*_*ν*_30*k*__

At level 1, scores are expressed as a function of so-called indicator variables. These variables indicate to which test the dependent variable refers. If the indicator variable *z*(1) is 1, the dependent variable test score refers to Test 1, if *z*(2) is 1, the variable test score refers to test 2, etc. A similar method using indicator variables for multivariate data analysis has been described before [25, 26].

At level 2, the effects of a participant’s background variables, that is, age, gender and educational background, are introduced. The level 2 model also includes the error terms *ϵ*_*ijk*_, which denote deviations of an individual’s observed test scores to that predicted by the model for that particular study.

At level 3, differences between studies are introduced by adding error terms *ν* to the intercept of each test. Note that the effects of age, gender and educational background are constrained to be the same in different studies, as it is unlikely that these effects differ between studies. This constraint can however easily be relaxed by adding error terms to those effects as well.

Substituting level 3 into level 2, and level 2 into level 1 yields the combined model (cf. Table 3). In this model, *γ*_100_ denotes the intercept of the first test. The interpretation of intercepts is dependent on the scaling of background variables. If age and education are centered on their mean and gender is contrast coded, the intercept *γ*_100_ refers to the scores on the first test for an “average” participant: of average age, not of a specific gender, with an average educational background. The parameters *γ*_110_, *γ*_120_ and *γ*_130_ denote the effects of age, gender and educational background on the first test. In addition to these so-called fixed effects, the model also yields estimators of random effects: the covariance matrix of within study errors *ϵ* and the covariance matrix of between study errors *ν*.

No constraints were imposed on the covariance structure of within study errors *ϵ* (cf. Table 3). Modeling each of the covariances between variables separately can account for both dependencies between variables within tests, and between variables that belong to different tests. Also, measurements can freely covary both positively and negatively. The covariance matrix of within study errors was constrained to be equal over studies, as is common in multilevel modeling [19].

As it is unlikely that test scores of “average” participants covary at the between study level, we imposed the constraint that between study errors *ν* did not covary (cf. Table 3). This constraint could be relaxed by adding these covariances to the model as well.

As mentioned in the introduction, one of the advantages of multilevel modeling is the handling of missing values. More specifically, multilevel models do not require that every participant has completed an equal number of tests. Multilevel models can be estimated with Full Information Maximum Likelihood (FIML) which uses all available information from each case [27, 28]. For FIML to result in correct parameter estimates, the missing data mechanism should be ignorable, i.e., the fact that an observation is missing should not be due to the value of that particular observation [18]. In the present case, missing data is due to the study design [29], and not due to the values of test scores that participants achieve. Since the participants that are pooled are all healthy, and the tests can be completed easily by healthy participants, missing data within studies should not occur systematically. Therefore, the missing data mechanism can be classified as ignorable, and FIML will yield adequate estimates.

In sum, multilevel modeling can be used to combine the results of multiple studies, even if data are missing, and it can incorporate background variables. Next, we indicate how multilevel models can be combined with multivariate normative comparisons to analyze whether an individual deviates from a composite normative database.

The multivariate normative comparison uses a version of Hotelling’s *T*^2^ statistic that is adapted for normative comparisons. If there are no background variables, the equation for this $T_{norm}^{2}$ is [7, 8]:

$$\begin{array}{c} T_{norm}^{2}=\frac{1}{(n+1)/n}\frac{n-p}{(n-1)p}{\left(\bar{y}-x\right)}^{\prime }C^{-1}\left(\bar{y}-x\right) \end{array}$$ (1)

where *n* is the number of participants in the norm group, *p* is the number of tests, $\bar{y}$ is a vector of length *p* containing the mean scores for every test in the norm group, *x* is a vector of length *p* containing the patient scores for every test, prime (′) denotes transposition, *C* is the *p*X*p* covariance matrix of the test scores in the norm group, and *C*^−1^ is the inverse of this covariance matrix.

Looking up $T_{norm}^{2}$ in the F-distribution with *p* numerator degrees of freedom, and *n*–*p* denominator degrees of freedom, yields a p-value corresponding to the probability that the patient would obtain this profile of scores (or a more extreme one) if he belongs to the same population as the norm group [8]; [7]. If this probability is very small, for example smaller than 0.05, the patient’s profile of scores is said to be deviating.

This normative comparison is two-sided, as both overall positive and overall negative deviations are considered abnormal. A one-sided variant has also been developed [7, 30]. In one-sided testing, all tests have to be standardized to bring them on the same scale. It is then decided that an individual is deviating from the norm if two conditions are satisfied: (1) the sum of deviations over tests is in the expected direction, and (2) the p-value does not exceed 0.10.

To account for the multilevel structure in the normative database, we make three adjustments to the multivariate normative comparisons method. First, the covariance matrix *C* is now the sum of two covariance matrices: the within study covariance matrix and the between study covariance matrix. Second, $\bar{y}$ now denotes the normative scores predicted given an age, gender and level of education that matches that of the patient. Third, the degrees of freedom have to be adjusted, as, in case of missing data, participants do not contribute information to the estimation of all parameters, and as individuals are nested within studies and thus observations are not completely independent [19].

There is no consensus on how degrees of freedom should be computed and different software packages use different methods [31]. We use the method implemented in the multilevel modeling software package *nlme* [32], which for our case equals the number of observations—(number of studies + number of estimated effects + 1).

Similar to the issue of determination of degrees of freedom, determination of the *n* to be used in Eq (1) is not straightforward when dealing with nested and missing data. Fortunately, once *n* becomes moderately large (above 100), even large differences in choice of *n* are of little influence. We set *n* equal to the total number of participants.

## Simulations

In simulation study 1, we investigated the effect of ignoring between study variance on the false positive rate. We did this by fitting models both with and without between study variance. In simulation study 2, we investigated the effect of missing data on false positive rate and sensitivity. In simulation 2, scores on certain tests were deleted for all participants in a study, as if the researchers in that study had decided not to administer that test.

### Methods

**Fig 2:**
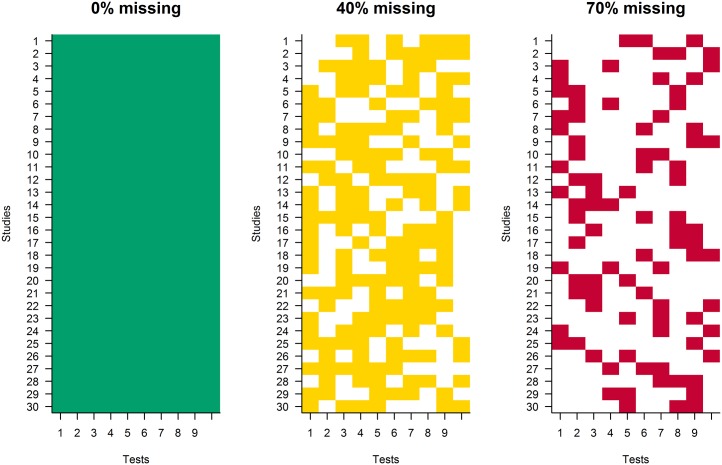
Missing data patterns for the 0%, 40% and 70% missing data conditions, with studies on the y-axis and tests on the x-axis. Colored boxes are non-missing test scores, white boxes are missing test scores.

The settings for the simulation studies are given in Table 4. In simulation study 2, either 0%, 40% or 70% of the data was made missing. Missing data was introduced by deleting data according to the pattern in Fig 2. The 0% condition is intended not as a control condition, but as a check of multilevel normative comparisons, without the added complication of missing data. Because of the nature of the aggregate database, 0% missing data will never be encountered in real settings. If only regularly administered tests were included in the database, only 40% to 70% missing data should be achievable. However, if all possible neuropsychological tests were included, the percentage missing test scores should be much higher; this would not allow the current model specification and normative comparison methods. This limitation is discussed further in the discussion section. Ten tests were used in the simulation: Twelve tests is the average number of tests that a neuropsychologist uses [33].

**Table 4:** Settings used in the two simulation studies.

Settings	
Number of tests	10
Number of participants per study	50
Number of studies	30
Percentage of test scores missing	Simulation 1: 0%
	Simulation 2: 0%, 40% or 70%
Number of simulations	1000 per condition

The parameter values for the two simulation studies are given in Table 5. The ANDI database was used to set the sample sizes of studies and the number of studies. The ANDI database was also used to estimate the effect sex, age and level of education would have on test scores. The simulation settings (see https://doi.org/10.5281/zenodo.321858) were based on these estimates. Information on the ANDI database (which groups contributed, how many studies and participants are available per test variable etc.) is presented in the documentation on www.andi.nl. Another large Dutch sample was examined to verify that effects as observed in the ANDI database can be considered representative [34]. The effects of background variables were all assumed to be linear. A parameter of −0.125 for age indicates for example that for every year that a participant increases in age, the participant on average achieves a score that is 0.125 points lower. The variance between studies was assumed to be small compared to the variance between participants within studies.

**Table 5:** Parameter values in the two simulation studies.

Parameters	
Intercepts	20
Age effect	−0.125
Gender effect	0.5
Education effect	1.25
Residual variance of test scores within studies	25
Residual correlation between test scores within studies	0.4
Residual variance of test scores between studies	5
Residual correlation between test scores between studies	0.0

In both simulation studies, patient data were simulated with the same parameters that were used to simulate normative data, on the understanding that patients’ scores differed from the scores in the norm group on 0, 1, 2, 5 or 9 tests. These deviations were introduced by subtracting two standard deviations (computed from the total variance) of the test scores in the norm group from the patient’s simulated test scores. So if patients truly deviated, they did so in a negative way. Two standard deviations could be considered the difference between patients and the norm group that is maximally interesting from a statistical perspective: Patients with much more extreme scores are easily recognized as being deviating, and patients with much less extreme scores are probably non-deviating. A 2 standard deviation difference is however a large difference in neuropsychological terms. Research has shown that 1 and 1.5 standard deviations are common for impairments that are secondary to a particular disorder, for example for attention problems that accompany major depression [35].

In applying the multivariate comparison, false positive rate was defined as the fraction of simulations in which a significant multivariate difference was observed in conditions in which there were no simulated differences. Sensitivity was defined as the fraction of simulations in which a multivariate difference was observed, in conditions in which simulated differences were present.

The multivariate results were contrasted with results of univariate comparisons. For the univariate comparisons, we recorded whether *any* of the patients’ scores, using an alpha of 0.05, deviated significantly from the norm univariately. This implies that in the power condition, we did not require that the deviation corresponded to any of the simulated deviations. This definition keeps results comparable between univariate and multivariate results, but works in favor of univariate comparisons: They do not need to be correct to be sensitive.

In the case of no simulated deviations, the rate of finding at least one deviation is known as the familywise error rate. It has been shown that the familywise error rate becomes much too high if multiple comparisons are made [7]. Therefore, corrections can be applied, such as the Bonferroni correction, which divides the criterion for significance by the number of comparisons. Therefore, we compared the results that were obtained using the multivariate comparisons to univariate comparisons that were either uncorrected, or Bonferroni corrected.

All comparisons were one-sided, as clinicians are generally only interested in patients’ performance being worse than in the norm group. This means that we used a p-value of 0.10 for the multivariate comparison as our criterion value, with the added criterion that the summed difference is in the expected direction, as described in the method section. Given these two criteria, we expect the overall proportion of significant deviations to equal 0.05 if no differences were simulated. For the univariate comparison, we used 0.05 as our one-sided criterion.

A critical p-value of 0.05 or equivalently a 95% confidence interval is often used in scientific research, but not in clinical practice. In clinical practice, more lenient criteria, such as 1 SD or 1.5 SD below the mean are common. In fact, research has shown that sensitivity and specificity may be optimal with such a 1.5 SD criterion [36]. However, in applications of the multivariate normative comparison, the 95% confidence interval has been shown to be sensitive to deviations, even in comparison to univariate results with more lenient criteria [9]. Therefore, the 0.05 criterion was used in these simulations as well.

We fitted the multilevel models using the software package *nlme* [32], because it is flexible in specifying covariance structures both for the *ϵ* and *ν* terms. *R* code that can be used to perform the entire analysis including the multivariate normative comparison can be found in the supporting information (S1 R Code).

## Results

### Simulation study 1

If between study variance was neglected, the false positive rate was 0.066 for the multivariate comparison, which is only slightly elevated compared to the required 0.05. If between study variance was estimated, the false positive rate was adequate, 0.050. For Bonferroni corrected univariate comparisons, the familywise false positive rate was 0.049 without estimated between study variance, and 0.047 with estimated between study variance. For uncorrected tests, the familywise error rate was too high; 0.306 without estimated between study variance, 0.276 with estimated between study variance.

### Simulation study 2

If 0% of the data were missing, the false positive rate was 0.060 for the multivariate comparison. If 40% of the data were missing, it was 0.059, whereas it was 0.097 if 70% of the data were missing. For the uncorrected univariate comparisons, the familywise error rate was too high, around 0.3, for all three percentages missing. For the Bonferroni corrected univariate comparison, the familywise error rate was 0.046, 0.046, and 0.040 for 0%, 40% and 70% missing. The multivariate results show that false positive rate is not completely under control if the percentage missing test scores becomes very high.

**Fig 3:**
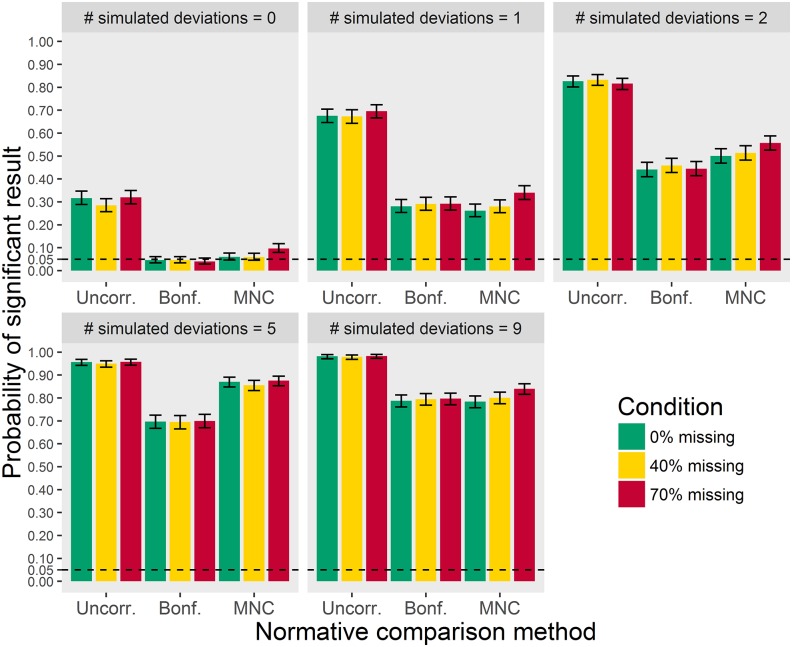
False positives (where number of deviations = 0) and sensitivity (where number of deviations > 0) as a function of the number of simulated deviations, for 0%, 40% and 70% missing data in the norm group. Error bars represent 95% confidence intervals.

With respect to power, as can be seen in Fig 3, uncorrected univariate comparisons show more significant results than multivariate or Bonferroni corrected univariate normative comparisons. Because familywise error was too high for uncorrected comparisons, the advantage in terms of power cannot be interpreted. Multivariate normative comparisons and Bonferroni corrected univariate comparisons show similar results in all conditions, with the exception of the 5 simulated deviations condition. When the patient deviates on 5 tests, the multivariate comparison is more sensitive.

Fig 3 also shows that sensitivity was about equal with 0% and 40% missing data. The comparisons with 70% missing data had slightly higher sensitivity. This should not be taken to suggest that 70% missing data is preferable, as the false positive rate was also slightly higher.

### Follow-up simulation studies

As a follow-up, we investigated the effect of the magnitude of between study variance on the false positive rate. To this end we computed the intraclass correlation (ICC), which is defined as the ratio of the between study variance and the sum of the within and between study variance. In simulation studies 1 and 2 this ICC was 0.167. A preliminary analysis of the ANDI database shows ICCs ranging from 0 to 0.4, depending on the type of tests under study. These ICC’s thus suggest that between study variance might vary considerably in real applications.

To investigate whether a larger between study variance affects false positive rate, we repeated simulation study 2 with a between study variance of 17, yielding an ICC of 0.4 (17 / (17 + 25)). With this higher level of between study variance, the false positive rates for multivariate normative comparisons were 0.060, 0.069 and 0.114 in the 0%, 40% and 70% missing data conditions. For Bonferroni corrected univariate normative comparisons, the false positive rates for these conditions were 0.060, 0.066 and 0.074. For uncorrected univariate comparisons, the false positive rates were too high, around 0.35. These results indicate that false positive rate only slightly increases if between study variance increases.

In realistic settings, not every study will have the same number of participants, i.e. sample sizes will be unbalanced. To investigate its effects, we ran simulations with a mean of N = 50, and a standard deviation of 10, with 70% missing data. These simulations showed a false positive rate of 0.112 for the multivariate comparisons, which is about the same as for the equal sample size case. Univariate uncorrected results showed a false positive rate of 0.302, while Bonferroni corrected results showed a false positive rate of 0.06. Therefore, unequal sample sizes do not seem to be problematic for multivariate or univariate comparisons. We also looked at simulations with unequal sample sizes and fewer participants, i.e a mean of N = 25, and a standard deviation of 5. For these simulations, the multivariate comparisons showed a false positive rate of 0.192, while the univariate uncorrected result was 0.327 and the Bonferroni corrected result was 0.054. The false positive rate is increased for the multivariate result. This seems to be because the problems of 70% missing data are combined with a mean decrease of 50% of the number of participants in this condition.

All simulations so far have been done with ten tests. We also checked whether the same results were obtained for 20 and 5 tests. Fitting models to data from 20 tests took considerably more resources than fitting models with 10 tests. Therefore, we only ran the 70% missing condition, and performed 100 rather than 1000 simulations. With 5 tests, we ran 1000 simulations with a 60% missing condition, as 70% of 5 does not give a whole number of test scores to remove.

A total of 11 simulations with 20 tests showed convergence issues and had to be rerun, demonstrating that with more parameters, results can become more unstable with this amount of missing data. Multivariate results showed a false positive rate of 0.17. Uncorrected univariate results showed a false positive rate of 0.36. Bonferroni corrected results showed a false positive rate of 0.02. The elevated type 1 error rate for multivariate comparisons seems to originate in less precise estimates of covariances between tests: Because the number of participants and studies were kept equal, increasing the number of tests implies that the number of studies in which two tests are administered together decreases. So some covariance estimates are based on a single study with 30 participants. Because the Bonferroni corrected tests do not use covariance, they remain conservative.

With 5 tests and 60% missing, the false positive rate was 0.07 for the multivariate comparisons. Uncorrected univariate results showed a false positive rate of 0.214. Bonferroni corrected results showed a false positive rate of 0.055. This shows that with fewer tests, the multivariate method performs appropriately.

Lastly, we investigated the effect of including fewer studies, as fewer than 30 studies might be available for some neuropsychological tests. We simulated data with 20 studies for 10 tests with 40% missing data, because with 70% missing not all covariances could be estimated. The false positive rate was 0.07 for the multivariate method, 0.326 for the univariate uncorrected method and 0.055 for the Bonferroni corrected method. So although the number of studies, and therefore also participants, was cut by a third, false positives rates were not affected.

### Empirical example

To give an impression of what the analysis would look like in practice, the method was applied to the ANDI database described earlier, and was used to examine the profile of a patient with Parkinson’s disease. The details of the Parkinson’s disease dataset have been described elsewhere [13, 37].

Because the ANDI database contains many tests, we only selected tests that the patient had completed, and fitted the model to only those tests. For this example, the model was fitted to two variables of the Auditory Verbal Learning Test (AVLT), three variables of the Stroop test, two variables of the Trail Making Test (TMT), one variable of the Letter Fluency Test, one variable of the Semantic Fluency Test, summing up to a total of nine variables. For each of these variables, more than 1700 participants were available in the ANDI database (www.andi.nl/home). All variables were demographically corrected for age, sex and level of education, except for TMT part A, for which correction for sex was not necessary. All test variables were transformed to normality using Box-Cox transformations, and were recoded and standardized [38].

**Fig 4:**
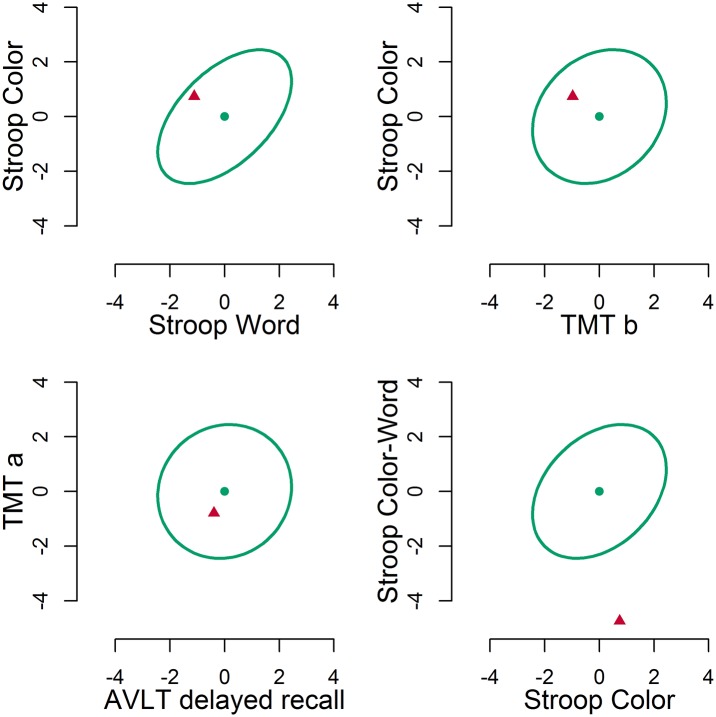
Four selected bivariate plots. The ellipses denote the 95% multivariate region. The dots denote the mean score in the norm group. The triangles depict the patient’s scores.

In Fig 4, four bivariate plots are given for the patient with Parkinson’s disease. A selection of two-dimensional plots is given because although the multivariate comparison provides a single result for eleven dimensions, this eleven-dimensional result is not easily visualized. As can be seen, correlations between variables differ, i.e. the shape of the bivariate distribution differs. The Stroop Color and Word variables in the top left plot are correlated, presumably because they belong to the same test and tap into the same naming speed component. The Stroop Color and TMT part b variables in the top right plot are only slightly correlated, presumably because although they both involve speed, one involves paper-and-pencil tracing, while the other involves verbal naming. Recalling words from memory after 30 minutes in the AVLT, and tracing a path in the TMT in the bottom left plot are completely uncorrelated, which is why the ellipse is circular. In all these bivariate plots, the patient falls within the 95% confidence interval. For the bottom right plot, this is not the case, as the patient falls far below the ellipse. This is mainly due to a very slow performance on the color-word interference condition of the Stroop. This slow performance is incongruent with the normal performance on the other Stroop subtask.

The multivariate test result is $T_{norm}^{2}(9,30902)=4.32$, p < 0.001. Using the one-sided criterion, we first have to ascertain whether the sum of differences is negative, which it is, −0.76. Therefore, we can conclude that this patient is impaired, as p <0.10.

## Discussion

Multivariate normative comparisons are a valuable tool in neuropsychological assessment. Therefore, it is important that a multivariate normative database becomes available. We proposed the construction of such a multivariate database by joining healthy control group data from published neuropsychological studies. In this paper we also outlined a solution to three issues that arise when constructing such a combined database. First, test scores may differ between studies. Second, not all tests are administered in all studies. Third, patients should be compared to controls of a similar age, gender and level of education. We developed a method that uses multilevel modeling to solve these three issues.

Our first set of simulations shows that estimating the variance between studies keeps false positive rate at an acceptable level. The results of our second set of simulations show that the number of false positives is too high if the percentage of missing data is 70%, but is satisfactory if 40% of the data is missing. Sensitivity of normative comparisons remains intact, even if 70% of the data is missing.

The power advantage, or enhanced sensitivity, of the multivariate comparison over Bonferroni corrected univariate comparisons was not visible in all conditions. Only when the patient deviated on half the tests, did the multivariate comparisons outperform Bonferroni corrected univariate comparisons. This is in line with earlier results [7], where it was shown that the advantage of the multivariate comparisons over univariate comparisons is greatest with intermediate numbers of deviations, with smaller advantages when the number of deviations is either very high or very low.

In the simulations with 20 tests and the simulations with smaller N, the false positive rate was not under control for the multivariate comparisons. This may be the result of the very large number of parameters needed in comparison to the number of participants, which primarily affected the estimates of the covariance between tests. A potential solution for such cases, if extra data collection is impossible, could be to provide restrictions on the covariances using a factor model, or to include prior information on the covariances.

Note that the proposed multilevel approach estimates between study variance. An alternative way to aggregate data over studies is to assume that between study variance does not need to be estimated. This assumption might in some applications be required if not sufficient studies are available to estimate this between study variance component [39]. Fortunately, in neuropsychology, sufficient studies are available as many studies administer the same instruments. Another alternative is to estimate between study variance, but to refrain from using it in comparisons. This may be more in line with current practice, where norms are used from a single normative study. However, we see the possibility to include between study variance as an advantage, as it allows for generalization, whereas assuming that between study variance is zero does not allow for generalization to new studies and new cases [40].

The current approach requires several assumptions. First, the multilevel procedure assumes that all contributing studies have drawn random samples of healthy participants. At first sight, this assumption may not be met in neuropsychological studies. For example, some researchers will only draw random samples from one gender, e.g. women, because they are studying the effects of a particular disease that occurs predominantly in women, e.g. breast cancer. This matching will however be harmless to our assumption of random sampling, as the assumption pertains to the data after correction for age, gender and educational background. As another example, close acquaintances of patients are popular controls: They are typically from similar educational backgrounds as the patient population and are often willing to participate [41]. Again, the fact that background is similar does not seem to be problematic, as educational background is included in the model. Finally, some control samples cannot be presupposed to be from the healthy population, such as non-schizophrenic psychiatric patients or even abstinent non-Korsakoff alcoholics [42, 43]. These should not be included in the composite normative database.

Second, multilevel analysis assumes that the included studies are randomly sampled from a population of studies. In practice, all available studies that fit the inclusion criteria would be included, rather than taking a sample. Therefore, we argue that this assumption is likely to be met. Note that this is similar to a random-effects meta-analysis, where all studies, and not a random sample, on the effect under investigation are included.

Third, the current methodology may allow for missing data at the level of individual participants. This requires that the missing data mechanism can be considered ignorable. Fortunately, we do not expect many non-ignorable missing values in neuropsychological studies. Patients may find it difficult to complete test batteries, e.g. because of fatigue. Therefore, test batteries are designed to be not too demanding [1]. This implies that healthy participants often can complete the entire battery, and therefore few scores are generally missing. Because the number of non-ignorable missing data points, if present, should thus be small, the amount of bias in the estimates they incur will most likely be negligible.

Fourth, the normative comparison method assumes that scores are multivariate normally distributed around predicted scores. Little is known about the multivariate distribution of tests because large multivariate datasets have generally not been available. We do however know that violations of univariate normality, which preclude multivariate normality, are common in neuropsychology. Neuropsychological test scores may for example be skewed and truncated by ceiling or floor effects [44]. Statistical tests have been shown to be generally robust to mild violations of distributional assumptions in a group comparison setting [45] as well as in a normative comparison setting [46] but more serious violations may result in a larger false positive rate. The multivariate comparison method has been shown to be robust to varying levels of skewness of the multivariate distribution but not to varying levels of kurtosis [8]. Solutions that have been proposed when multivariate normality is not tenable, involve transformations of the data [47] or non-parametric comparisons [8].

Fifth, the current method requires calculation of the covariance between every pair of tests. Therefore, every test has to be administered with each of the other tests to at least a few participants. This limits the number of tests that can be included, as only the more common tests will have been administered together with all other tests. This was the case for the empirical example: A selection of tests had to be made to ensure that all covariances could be estimated with the present method. If less common tests need to be included, solutions may lie in models that restrict covariances, for example to obey a certain factor structure, or in collecting additional data [48].

The current approach can be extended in a variety of ways. First, although the proposed model flexibly handles missing data in test scores, it still resorts to listwise deletion of cases having a missing value on one of the covariates. Because missing covariates are handled differently from missing scores, this may result in many cases being dropped that were previously included. In these situations, alternatives to FIML such as multiple imputation might be a good solution.

This method can be extended beyond clinical neuropsychology, but note that clinical neuropsychology has three advantages that may not be present in every other field. The first is that neuropsychological test administration has been standardized to a high degree, such that data from different studies can be pooled. If there are for example differences between how tests are scored, additional steps may be necessary to harmonize measurements across studies [39]. The second advantage is that clinical neuropsychology is a large field, so many studies are available that have tested control groups. In smaller fields, it may be difficult to find sufficient studies that have administered the same test to accurately estimate between study variance. The third is that neuropsychologists administer multiple tests to the same participants, and therefore covariances between tests can be estimated. In fields where smaller test batteries are common, the lack of overlapping tests may imply that multivariate normative comparisons according to the current methodology are not feasible. These advantages are however present in other fields, for example, in personnel psychology where highly standardized tests are regularly administered in large batteries. But also outside of psychology, the methods described here can be used just as easily for example in medicine, where physiological measures like blood pressure and heart rate are compared against the norm. Profiles of such measures could be compared against the norm as well using the multivariate method described here.

In conclusion, a large composite multivariate normative dataset can be established by combining data from many different studies. The current multilevel extension of multivariate normative comparisons can be used to handle (i) variability in test scores between studies (ii) missing data which arise because not all studies administered the same tests, and (iii) background variables. This multilevel extension allows routine multivariate comparisons of patients’ test scores to multivariate normative data. This will enhance sensitivity of normative comparisons in neuropsychology, and may also be valuable in other contexts, e.g. in clinical or personnel psychology or medicine.

## Supporting information

S1 R Code Model fitting code & multivariate comparisons code. Requires the *nlme* package.
(DOCX)
