# Supplementary material for: Multivariate normative comparisons using an aggregated database
Source: PLoS One. 2017 Mar 7;12(3):e0173218. doi: 10.1371/journal.pone.0173218 (PMC5340373; doi:10.1371/journal.pone.0173218)
Supplement: S1 R Code — Requires the nlme package. (DOCX) [file pone.0173218.s001.docx]

**Supporting Information**

***Model fitting code***

*library(nlme)*

*fit <- lme( score ~ -1 + z1 + z2 + z3 + z1:age + z2:age + z3:age + z1:gender + z2:gender + z3:gender + z1:edu + z2:edu + z3:edu, correlation=corSymm(form = ~ test | study/ID), weights = varIdent( form =~ 1 | test), random = list(study = pdDiag(~ -1 + z1 + z2 + z3 )), data=dat)*

*lme fits a linear mixed model with the following arguments:*

1. *the first argument ("score ~ -1 + z1 + z2 + z3 + z1:age + z2:age + z3:age + z1:gender + z2:gender + z3:gender + z1:edu + z2:edu + z3:edu") describes the fixed effects, in which "score" is the dependent variable, "-1" suppresses the overall intercept, "z1", "z2" and "z3" describe the fixed intercepts per test, and the interaction effects "z1:age" to "z3:edu", describe fixed effects of the background variables per test.*
2. *the second argument ("correlation=corSymm(form = ~ test | study/ID)") describes the correlation structure of the error terms on level 1. The error terms are nested under "ID" (which is in turn nested under "study") and are indexed by "test". "corSymm" lets the correlation structure be completely free, so all correlations between tests are estimated. This corresponds to the covariance part of the "Unstructured" covariance structure.*
3. *the third argument ("weights = varIdent( form =~ 1 | test)") specifies the variance of the error terms. The variance of the error terms is unique to each level of "test", i.e. heteroscedastic. varIdent lets each of these be freely estimated. This corresponds to the variance part of the "Unstructured" covariance structure.*
4. *the fourth argument ("random = list(study = pdDiag(~ -1 + z1 + z2 + z3 ))") specifies the covariance structure of the between study random effects. The covariance between tests at the study level is fixed to 0, so the covariance matrix is diagonal ("pdDiag"). "-1" suppresses the overall random intercept, and "z1", "z2" and "z3" specify the random intercepts per test. This specifies the "Variance Components" covariance structure.*
5. *the fifth argument ("data = dat") specifies the used dataframe that is formatted like Table 2 in the method section.*

***Multivariate comparison code***

*extract.est.cov.matrix.within <- function( fit = fit, no.tests){*

*firstnum <- which(is.na(match(1:no.tests, sort(as.numeric(names(coef(fit$modelStruct$varStruct,unconstrained = FALSE)))))))*

*step1 <- rbind(as.numeric(c(1,coef(fit$modelStruct$varStruct,unconstrained=FALSE))),as.numeric(c(firstnum, names(coef(fit$modelStruct$varStruct,unconstrained = FALSE)))))*

*est.std.matrix.within <- fit$sigma*step1[,order(step1[2,])][1,]*

*est.cor.matrix.within <- matrix( NA, no.tests, no.tests)*

*est.cor.matrix.within[lower.tri(est.cor.matrix.within)] <- coef(fit$modelStruct$corStruct, unconstrained = FALSE)*

*est.cor.matrix.within <- t(est.cor.matrix.within)*

*est.cor.matrix.within[lower.tri(est.cor.matrix.within)] <- coef(fit$modelStruct$corStruct, unconstrained = FALSE)*

*diag(est.cor.matrix.within) <- 1*

*est.cov.matrix.within <- diag(est.std.matrix.within) %*% est.cor.matrix.within %*% diag(est.std.matrix.within)*

*return(est.cov.matrix.within)*

*}*

*obs.x <- patient.data$score*

*P <- length(obs.x)*

*N <- length(unique(dat$ID))*

*est.cov.matrix.between <- as.numeric(VarCorr(fit)[1:P,1])*diag(P)*

*est.cov.matrix.within <- extract.est.cov.matrix.within(fit, P)*

*C <- est.cov.matrix.within + est.cov.matrix.between*

*inv.C <- solve(C)*

*pred.y <- predict( fit, patient.data, level = 0)*

*Tsquared <- ( 1 / (( N + 1 ) / N )) * ( ( N - P ) / ( ( N - 1 ) * P ) ) * t( pred.y - obs.x ) %*% inv.C %*% ( pred.y - obs.x )*

*DF1 <- P*

*DF2 <- N - P*

*tailed <- "one-tailed (left)"*

*if( tailed == "one-tailed (left)"){*

*if( sum(pred.y - obs.x) > 0 ){*

*pvalue <- ( 1 - pf( Tsquared, DF1, DF2 ) ) / 2*

*} else { pvalue <- 1 }*

*}*

*if( tailed == "one-tailed (right)"){*

*if( sum(pred.y - obs.x) < 0 ){*

*pvalue <- ( 1 - pf( Tsquared, DF1, DF2 ) ) / 2*

*} else { pvalue <- 1 }*

*}*

*if( tailed == "two-tailed") {*

*pvalue <- 1 - pf( Tsquared, DF1, DF2 )*

*}*

*The first function ("extract.est.cov.matrix.within"), with the model object and number of tests as arguments, serves to extract the estimated within study covariance matrix from the model object. This requires a few lines of code, as the model object does not have the variances stored in the order we would want, and stores correlations instead of covariances. The estimated between study covariance matrix is easier to obtain, with the "VarCorr" function.*

*Noteworthy is the "predict" function, which calculates the predicted values for a person of the patient's age, gender and educational background, using the coefficients from the model object. The argument "level = 0" specifies that a new population prediction is needed, rather than that the patient's study has to be taken into account.*

*As noted in the main text, the choice of N, and therefore also the choice of numerator degrees of freedom is difficult. However, any reasonable choice of N is of little influence, as "( 1 / (( N + 1 ) / N )) * ( ( N - P ) / ( ( N - 1 ) * P ) )" is already quite close to its asymptote 1 / P at N = 200.*
